# Nitrogen-Drag-Assisted Crosslinking by Glucose to Obtain Bovine Serum Albumin–SGI-1027 Nanoparticles

**DOI:** 10.3390/pharmaceutics18101310

**Published:** 2026-10-15

**Authors:** Mercedes G. López, Laura E. Zavala-García, Ángela F. Kú-González, Alejandro Solís-Hernández, Ramón Carriles, Silvio Zaina, Lino Sánchez-Segura

**Affiliations:** 1 Departamento de Biotecnología y Bioquímica, Centro de Investigación y de Estudios Avanzados del Instituto Politécnico Nacional, Unidad Irapuato, Irapuato 36824, Guanajuato, Mexico; 2 Departamento de Ingeniería Genética, Centro de Investigación y de Estudios Avanzados del Instituto Politécnico Nacional, Unidad Irapuato, Irapuato 36824, Guanajuato, Mexico; 3 EPILABUG, Division of Health Sciences, Leon Campus, University of Guanajuato, León 37320, Guanajuato, Mexico; 4 Unidad de Biología Integrativa, Centro de Investigación Científica de Yucatán, Mérida 97200, Yucatán, Mexico; 5 Department of Medical Sciences, Division of Health Sciences, Leon Campus, University of Guanajuato, León 37320, Guanajuato, Mexico; 6 Centro de Investigaciones en Óptica A.C., León 37150, Guanajuato, Mexico

**Keywords:** atherosclerosis, crosslinking, SGI-1027, solvent drag, nanoparticle, Raman spectroscopy, cellular uptake

## Abstract

**Background/Objectives**: Nanoparticle (NP) technology is a promising tool against atherosclerosis, an inflammatory disease of large- and medium-sized arteries and an underlying cause of cardiovascular disease. Notably, DNA methyltransferase inhibitors such as SGI-1027 are anti-inflammatory. SGI-1027 tissue target specificity and potency may improve when loaded into an NP. We have previously shown that SGI-1027 nanoencapsulation with serum albumin NPs is anti-inflammatory in cell culture and anti-atherogenic in vivo. Nonetheless, nanoencapsulation relied on glutaraldehyde, which limits pharmacological applications. To increase NP biocompatibility, we assessed an alternative crosslinking method based on glucose and solvent drag to synthesize bovine serum albumin (BSA) NP loaded with SGI-1027. We assessed the physicochemical and functional parameters of BSA NPs loaded with increasing SGI-1027 doses (0, 0.05, and 0.1 mg). **Results**: We found that SGI-1027 encapsulation improves key NP properties in a SGI-1027-dose-dependent fashion: NP yield, drug encapsulation efficiency, morphology, and morphometrics; on the other hand, the ζ-potential and polydispersity were not affected. A molecular interaction between BSA and SGI-1027 was found at 1057–906 cm^−1^ using FT-IR. Raman spectroscopy revealed a fingerprint of SGI-1027 encapsulated into BSA at 728–650 cm^−1^, corresponding to the 2,4-diamino-6-methylpyrimidine of SGI-1027. NPs were internalized by mouse peritoneal macrophages, where accumulation at the cell membrane border, cytoplasm, and nuclear membrane was observed only when loaded with SGI-1027. Internalization coincided with an anti-inflammatory response. **Conclusions**: We found a synergistic effect between glucose and N_2_ (solvent drag) on protein self-assembly nanostructuration; this methodology offers an alternative to glutaraldehyde and heat crosslinking to encapsulate hydrophobic or thermolabile drugs.

## 1. Introduction

Nanoparticle (NP) technology promises to enhance drug potency and target specificity to improve prevention and therapy for a wide range of diseases, including cardiovascular disease (CVD), a leading cause of invalidity and mortality worldwide [1,2]. Atherosclerosis is an inflammatory disease of the arterial wall and a major underlying cause of CVD [3]. Inflammation is driven by both innate and adaptive immune cells, such as macrophages and T lymphocytes, respectively, in atherosclerosis [4]. Consequently, inflammation is considered a major target for the therapy of atherosclerosis. Several clinical trials with anti-cytokine antibodies or immunosuppressants (CANTOS, LoDoCo2, COLOT) have successfully proven that inflammation is causally linked to atherosclerosis but have been hampered by side effects such as immunosuppression increasing the infection and cancer risk and anemia, compounded with a short drug half-life in the circulation [5]. Additionally, DNA methylation inhibitors, including azacytidine and SGI-1027, have been shown to be anti-inflammatory [6]. Work by several groups, including ours, has shown the anti-atherogenic and anti-inflammatory activities of SGI-1027 in cultured cells and in mouse models [7,8]. SGI-1027 is a compound derived from quinoline that belongs to the family of non-nucleoside inhibitors of DNA methyltransferases [9]. The chemical structure of SGI-1027 is composed of four fragments of 4-aminoquinoline, 4-aminobenzoic acid, 1,4-phenylenediamine, and 2,4-diamino-6-methylpyrimidine linked in sequence with a para/para orientation [10]. The main hurdles with its application as a pharmacological treatment are extremely poor solubility due to high lipophilicity and lack of specificity for the vascular wall. To address these issues, the nanoencapsulation of SGI-1027 with human serum albumin (HSA) has shown improved water solubility and facilitated administration and intracellular uptake [7]. A crucial step of serum albumin encapsulation is crosslinking with glutaraldehyde. The effects of glutaraldehyde have been extensively studied, with an association between residual toxicity and crosslinked biomaterials [11,12,13]. In addition, glutaraldehyde has been shown to adversely affect the physicochemical properties of NPs, such as coalescence [14]. These findings demonstrate the need for glutaraldehyde-independent crosslinking methods.

The desolvation process, a key step in protein NP synthesis, consists of increasing the concentration of the desolvation agent in the dissolved solute (e.g., an aqueous albumin solution) over a short period [14,15,16]. In this step, albumin molecules undergo polycondensation, forming lower-order structures that assume a spherical shape by the effect of hydrodynamic forces in the reactor. Once the solute is nanostructured into colloidal particles in suspension, the subsequent stage is the stabilization of the NP by adding a crosslinking agent [17]. However, one aspect that should be considered critically in the desolvation process is the removal of solvent from the coacervated polymer [18]. This work aimed to develop a method for glucose crosslinking, assisted by solvent drag, to synthesize BSA NPs loaded with SGI-1027. We also assess the effects on the physicochemical and functional parameters of the NP.

## 2. Materials and Methods

### 2.1. Chemicals

The lyophilized powder of BSA (66 kDa) was purchased from Equitech-Bio, Kerrville, TX, USA. The SGI-1027 was pharmaceutical-grade (≥98% purity HPLC) (Sigma-Aldrich, St Louis, MS, USA). Other chemical reagents were dimethyl sulfoxide DMSO (≥99.5% purity) (Sigma-Aldrich, St Louis, MS, USA), sodium chloride (Merck, Darmstadt, Germany), absolute ethanol (≥99.8% purity) (Merck, Darmstadt, Germany), sodium dodecyl sulphate (SDS) (Sigma-Aldrich, St Louis, MS, USA), Coomassie Brilliant Blue R-250 (CBB) (Bio-Rad Laboratories, Inc., Kidlington, UK), glycerol (Merck, Darmstadt, Germany), and β-mercaptoethanol (Sigma-Aldrich, St Louis, MS, USA). For ethanol drag, nitrogen gas (≥99.9% purity) was used (INFRA, San Nicolás de los Garza, Mexico).

### 2.2. Synthesis of BSA NPs Loaded with SGI-1027

NP were prepared by adapting previously described albumin desolvation procedures [14,19,20]. BSA powder (120 mg) was first solubilized in 4 mL of previously sterilized deionized water (DW), and then, the pH was adjusted to 10 using NaOH powder. This formulation was agitated for 30 min at 1000 rpm with a Eurostar 20 stirrer (IKA, Wilmington, NC, USA) at ambient temperature. Then, 0, 50, and 100 µL of a stock solution of SGI-1027 [1 mg/mL] in DMSO were added to obtain final concentrations of 0, 0.05, and 0.1 mg, respectively. Protein desolvation was initiated while stirring at 1000 rpm by delivering 16 mL of ethanol at 1.5 mL/min through an NE-1060 syringe pump (New Era Pump System Inc., Farmingdale, NY, USA). Glucose was used as the stabilizing reagent, adapting the approach of Bartlett et al. [21]: 240 µL of a 15% glucose stock was introduced into the reactor while stirring. One minute later, N_2_ flow was started at 250 standard cubic centimeters per minute (SCCM) and maintained, together with agitation, for 7 h. Subsequently, the NPs were deposited into a 15 mL Falcon tube and maintained on a rotating platform at 45 rpm (MR1512 mini rotator, Glas-Col, Terre Haute, IN, USA) for 24 h. To compare synergistic crosslinking between glucose and N_2_, treatments of 0 mg SGI-1027 with glucose but N_2_-lacking and glucose-deprived with N_2_-injected were carried out. The NPs were washed via centrifugation at 12,485× *g* for 40 min at ambient temperature (MC-12V, DuPont, Newtown, CT, USA), and the pellet was resuspended in sterile DW at pH 7.0 in each cycle. The last pellet was resuspended in sterile DW using a vortex mixer (MX-S, SCILOGEX, Rocky Hill, CT, USA) and then stored at 4 °C.

### 2.3. Transmission Electron Microscopy (TEM) and Morphometric Analysis of NPs

In order to visualize the shape of NPs, they were examined using TEM (Morgagni M-268, Philips/FEI, Brno, Czech Republic). For grid preparation, 7 µL of each treatment was added to a 200-mesh copper grid cover with formvar/carbon (Ted Pella Inc., Redding, CA, USA) and allowed to stand for 7 min. Following a 7 min incubation, 2.0% uranyl acetate was used to contrast the samples (Electron Microscopy Science Inc., Hatfield, PA, USA), and further incubation was carried out for 10 min. For all experiments, the TEM operating settings were 80 kV high-voltage (EHT) and a vacuum pressure of 5 × 10^−5^ Torr. NP images were acquired at 22,000× for low magnification (shadow magnification) and 71,000×–140,000× for high magnification, respectively. Images were saved in .TIFF (Tagged Image File Format) with 1376 × 1032 pixels in greyscale. The 0 and 255 values were assigned to black and white, respectively, in the greyscale.

For quantitative morphometry, 50 individual particles from each treatment were isolated from the TEM fields. Each particle image was placed on a white background and standardized to 1376 × 1032 pixels as an 8-bit TIFF at a calibration of 2.1 pixels/nm. Measurements were obtained in ImageJ v1.49p (National Institutes of Health, Bethesda, MD, USA) using the shape_descriptor1u add-on. The area (*A*), effective diameter (*Ed)*, aspect ratio (*Ar*), and shape factor (*Sf*) were the measured particle descriptors. The *A* was quantified by the addition of pixels within a selection or a determined ROI, with optional conversion to calibrated units in the case that the image had a defined scale. *Ed* represents the diameter of an object approximated to a circle with the same area to the object being examined, following the Syverud et al. [22] equation (1). *Ar* represents the particle width:length ratio and was obtained as described in Parakhonskiy et al. [23]. *Ar* effectively describes particle ellipticity, as *Ar* < 1, *Ar* = 1, and *Ar* > 1 correspond to “flat”, circular and elliptical particles, respectively (*ibidem*, Equation (2)). The particle projected area and the overall projection perimeter (*P*) are used to calculate *Sf*—i.e., circularity—as proposed by Bouwman et al. [24] (Equation (3)).

(1) $$\mathrm{Ed}=\sqrt[{2}]{\frac{A}{\pi }}$$

(2) $$\mathrm{Ar}=\frac{\mathrm{Major axis}}{\mathrm{Minor axis}}$$

(3) $$\mathrm{Sf}=\frac{4\cdot \pi \cdot A}{P^{2}}$$

### 2.4. Quantification of BSA Transformed into NPs and Encapsulated SGI-1027

To determine the amount of encapsulated SGI-1027, the NPs were disassembled and the recovery was performed in DMSO, a solvent with higher SGI-1027 solubility; 500 μL of NPs was sedimented at 12,485× *g* for 15 min and resuspended with sterile DW, repeated three times at ambient temperature; the supernatants were discarded. The samples were lyophilized (HETO MAXY dry, LYO, Lillerød, Denmark) and broken by adding 100 µL of DMSO. The NPs were homogenized for 1 min (MX-S, SCILOGEX, Rocky Hill, CT, USA), sonicated for 30 min at 35 °C, and incubated for 12 h at 4 °C. Subsequently, the NPs were sonicated for 30 min at 35 °C and centrifuged at 12,485× *g* for 30 min (MC-12V, DuPont, Newtown, CT, USA). The supernatants were directly deposited in HPLC vials and stored at −20 °C. Denaturalized proteins were weighed in an analytical balance (Ohaus Analytical Plus, Pioneer, Shanghai, China). BSA NP yields were calculated using equations described by Bhalekar et al. [25] and Bartlett et al. [21]. The assessment of BSA nanostructured into NPs was updated to the total volume recovered from the NP synthesis. The proposed Equation (4) was calculated for each treatment as follows:

(4) BSA NPs yield%=BSA in NP mginitial BSA mg× 100

The encapsulated SGI-1027 was quantified via HPLC-UFLC (SPD-20A, Shimadzu, Camby, OR, USA). The identification of SGI-1027 was reached using a Shimadzu Shim-pack GIST C18 column with a 100 Å pore size (3 μm, 4.6 mm × 50 mm) (Part number: 227-30011-03, Shimadzu) and reverse phase consisting of methanol (A) and 0.1% trifluoroacetic acid/water (B) [A:B (40:60, *v*/*v*)] in isocratic method at a flow rate of 1.0 mL/min. The absorbance was measured at 360 nm, and ultraviolet (UV) spectra were recorded over 220–350 nm at an acquisition rate of 1.25 scans/s. Reference curves were constructed using several concentrations (0, 1, 2, 3, 4, 5, 6, 8, 10, 20, 30, and 50 ng/mL) of the SGI-1027 standard. The curve and samples were injected with 1 μL and 20 μL of the solutions, respectively, both in triplicate. To determine the encapsulation efficiency (EE) (%), total SGI-1027 loaded into NPs was estimated from the total volume recovered at the end of the NP synthesis. The EE (%) was calculated with Equation (5) as follows:

(5) $$\mathrm{Encapsulated}\;\mathrm{efficiency}\;\left(\%\right)=\frac{\mathrm{encapsulated SGI}-1027(\mathrm{mg})}{\mathrm{initial SGI}-1027(\mathrm{mg})}\times 100$$

### 2.5. Measurement of Particle Size, Polydispersivity Index, and ζ-Potential

The hydrodynamic size, polydispersity index (PDI), and ζ-potential were characterized with a Zetasizer Nano ZS-90P analyzer (Malvern Instruments, Worcester, UK). Before size analysis, purified NP suspensions were diluted 20-fold with DW, transferred to a particle-size analytical cuvette (DTS0012, Malvern Instruments), and measured at a 12.8° scattering angle at 25 °C. The Smoluchowski equation was used to calculate data, where a ≈100 nm particle size greatly exceeds the Debye length of ≈1 nm [26]. The ζ-potential was measured in a ζ-potential-dedicated cuvette (DTS1070, Malvern Instruments). SigmaPlot 12 (Systat Software, Inc. Sausalito, CA, USA) was used to analyze ζ-potential data.

### 2.6. Fourier Transform Infrared (FTIR) Spectroscopy

Identification and analysis of functional groups in the interaction between BSA and SGI-1027 were performed using an iS50 Nicolet FTIR spectrometer (Thermo Scientific, Madison, WI, USA) equipped with an attenuated total reflectance (ATR) diamond array. The spectra were acquired from lyophilized samples scanned at a wavenumber range of 4000–500 cm^−1^ in transmittance mode [%T]. SGI-1027 and BSA standards were analyzed directly, whereas NP samples were purified and lyophilized as aforementioned. Each spectrum comprised 64 scans at a nominal resolution of 4 cm^−1^, with air used as the single-beam. Three independently acquired spectra per sample were averaged. Spectral data were processed using OMNIC 9.13.1229 (Thermo Fisher Scientific Inc., Waltham, MA, USA). The reconstructed spectra were analyzed using SigmaPlot 12 (Systat Software, Inc. Sausalito, CA, USA).

### 2.7. Raman Spectroscopy

Raman measurements were obtained with DXR3xi Raman Imaging Microscope (Thermo Fisher Scientific, Waltham, MA, USA) operated in conventional analysis mode. The operation conditions were BSA analysis at an excitation wavelength of 532 nm, with a laser power of 8.1 mW; SGI-1027 was 1.0 mW, with an exposure time of 1 s. Spectra covered 3500–100 cm^−1^. Powdered BSA and SGI-1027 standards were homogenized, deposited on glass slides and analyzed directly. Prior to analysis, the BSA NPs loaded with SGI-1027 were washed and lyophilized as mentioned above. The samples were resolved and analyzed with a 50×/1.4, NA ∞−0.17, Olympus Plan FLUAR objective. Three spectra were collected for every sample and averaged to generate the spectrum used for analysis. For every sample, three replicates were averaged to obtain the average spectrum. Spectra were decoded in OMNIC 9.13.1229 (Thermo Fisher Scientific Inc., Waltham, MA, USA). The reconstructed spectra were analyzed using SigmaPlot 12 (Systat Software, Inc. Sausalito, CA, USA).

### 2.8. Sodium Dodecyl Sulphate–Polyacrylamide Gel Electrophoresis (SDS-PAGE)

The molecular-mass distribution of BSA released from NPs containing 0, 0.05, or 0.1 mg of SGI-1027 was examined via reducing SDS-PAGE based on the Laemmli Tris-glycine system [27], with modifications from Carriles et al. [28]. An EZ-Run prestained recombinant protein ladder spanning 10–170 kDa (Fisher Scientific, Waltham, MA, USA) provided molecular-mass bands as a reference. Raw BSA [4 mg/mL] and the three NP preparations were mixed with sample buffer containing 50 mM Tris–HCl (pH 6.8), 2% SDS, 0.1% CBB, 10% glycerol, and 100 mM β-mercaptoethanol and heated for 5 min. Aliquots of 20 µL were deposited onto gels containing a 4% stacking layer and a 12% resolving layer. Electrophoresis was run at 60 V during stacking and 100 V during separation. Gels were rinsed with DW for 5 min and subsequently stained with CBB solution (0.025% Coomassie dye, G-250 in 10% acetic acid). After an additional wash with DW, gels were incubated for 12 h under agitation and clarified to improve band visualization. Each treatment was represented by three experimental replicates per gel. Images were recorded at 600 dpi with a Gel Doc^TM^ XR (Bio-Rad Documentation Systems) with a digital resolution of 1580 × 1489 pixels in RGB (Red, Green, and Blue) and .TIFF.

### 2.9. Cellular Uptake of BSA NPs Loaded with SGI-1027 by Mouse Peritoneal Macrophages

C57BL/6 mice were obtained from the stock bred at the animal house facility of the Division of Health Sciences, Leon Campus of the University of Guanajuato, Mexico. Mice were fed ad libitum with a standard chow (diet 5008, LabDiet, St. Louis, MO, USA) and were under continuous health supervision by the Veterinarian assigned by the University of Guanajuato. Mouse peritoneal macrophages (MPMs) were obtained from three 8–9-week-old females via peritoneal washing, essentially as described by De Jesus et al. [29]. MPMs from each mouse were centrifuged at 500× *g* for 10 min, and the cell pellet was resuspended in 4 mL of culture medium (RPMI-1640, Gibco, Grand Island, NY, USA), 2 mM L-Glutamine (Sigma-Aldrich, St Louis, MS, USA), 10% Foetal Calf Serum (Thermo Fisher Scientific, Waltham, MA, USA), and 1% Penicillin/Streptomycin (Thermo Fisher Scientific, Waltham, MA, USA). From each mouse (3 individuals), we obtained four dishes, for a total of twelve dishes. We plated approximately 2.5 × 10^5^ cells per dish and allowed them to adhere for 2 h. Culture conditions were maintained at 37 °C and 5% CO_2_ throughout the procedure. Medium containing non-adhering cells was discarded. Fresh medium was added to adhering MPMs and administered NPs at 0, 0.05, and 0.1 mg of SGI-1027, previously washed and stained with eosin Y as reported by Márquez-Sánchez et al. [7]. A control was administered 100 µL of water (negative control). Incubation with NPs or the control was carried out for 24 h. Therefore, the administration of NPs was performed in experimental triplicate, without technical replications. The experiments with cells and animals reported in this research were approved by the Institutional Care and Use of Laboratory Animals Committee (CICUAL) of the University of Guanajuato, protocol number CEPIUG-P36-2023.

### 2.10. Inflammatory Cytokine Secretion and Multiphoton/Confocal Microscopy Analysis

At the end of the incubation of MPMs with NPs or the control, cell culture medium was obtained and cells were fixed for cytokine determination and microscopy, respectively. A panel of cytokines (interleukin-1β (IL-1β), IL-6, IL-8, and interferon α (IFNα)) was determined via an immunocapture-based quantitative PCR multiplex assay (qPCR; PreciseDX Lab, Chihuahua, Chi., Mexico). MPM cell fixation was carried out via incubation in 4% paraformaldehyde freshly dissolved in 0.16 M phosphate buffer (PB) for 12 h at 4 °C. The fixed MPM cells were washed three times with PB buffer and stained with 2.5 µL of FM4-64 from a stock solution of FM4-64 [5 mg/mL] dissolved in sterile DW and incubated for 1.5 h at 4 °C. The second staining consisted of a mixture of 7.5 µL of DAPI (Sigma-Aldrich, St. Louis, MO, USA) dissolved in 500 µL of sterile DW. Finally, the MPM cells were washed with distilled water. Visualization was carried out using a multiphoton microscope (LSM 880-NLO, Zeiss, Oberkochen, Germany) coupled to an infrared Ti: Sapphire laser (Chameleon Vision II, COHERENT, Santa Clara, CA, USA). The operational parameters for visualizing all treatments were an argon laser at 488 nm at 1.0% power and 543 nm at 0.3% power, a Chameleon laser at 760 nm at 1.8% power, and a completely open pinhole. The samples were scanned with a water immersion objective (40X/1.0, DIC VIS-IR ∞−0.17, Zeiss W Plan-Apochromat). The channels blue, yellow, and red that compose the images correspond to DAPI (371–476 nm) for the nucleus, Alexa 488 (526–579) for stained NPs with eosin Y, and FM4-64 (650–745 nm) for the cell membrane, respectively. All micrographs were acquired in CZI format at 2048 × 2048 pixels and RGB.

### 2.11. Data Analysis

NP synthesis and physicochemical characterization were repeated in at least three independent experiments. Summary values are reported as the mean ± SD. Data were analyzed using an ANOVA One Way. When the omnibus comparison indicated differences among groups, Student–Newman–Keuls multiple comparisons were used to identify the contributing groups. For some parameters, group distributions were analyzed non-parametrically, and treatment effects were tested with the Kruskal–Wallis procedure. A *p* value ≤ 0.05 was considered statistically significant. Data of pro-inflammatory cytokines were analyzed using an ANOVA and Scheffé post hoc test for performing multiple pairwise comparisons.

## 3. Results

### 3.1. Reactor and Drag System Using N_2_

**Figure 1:**
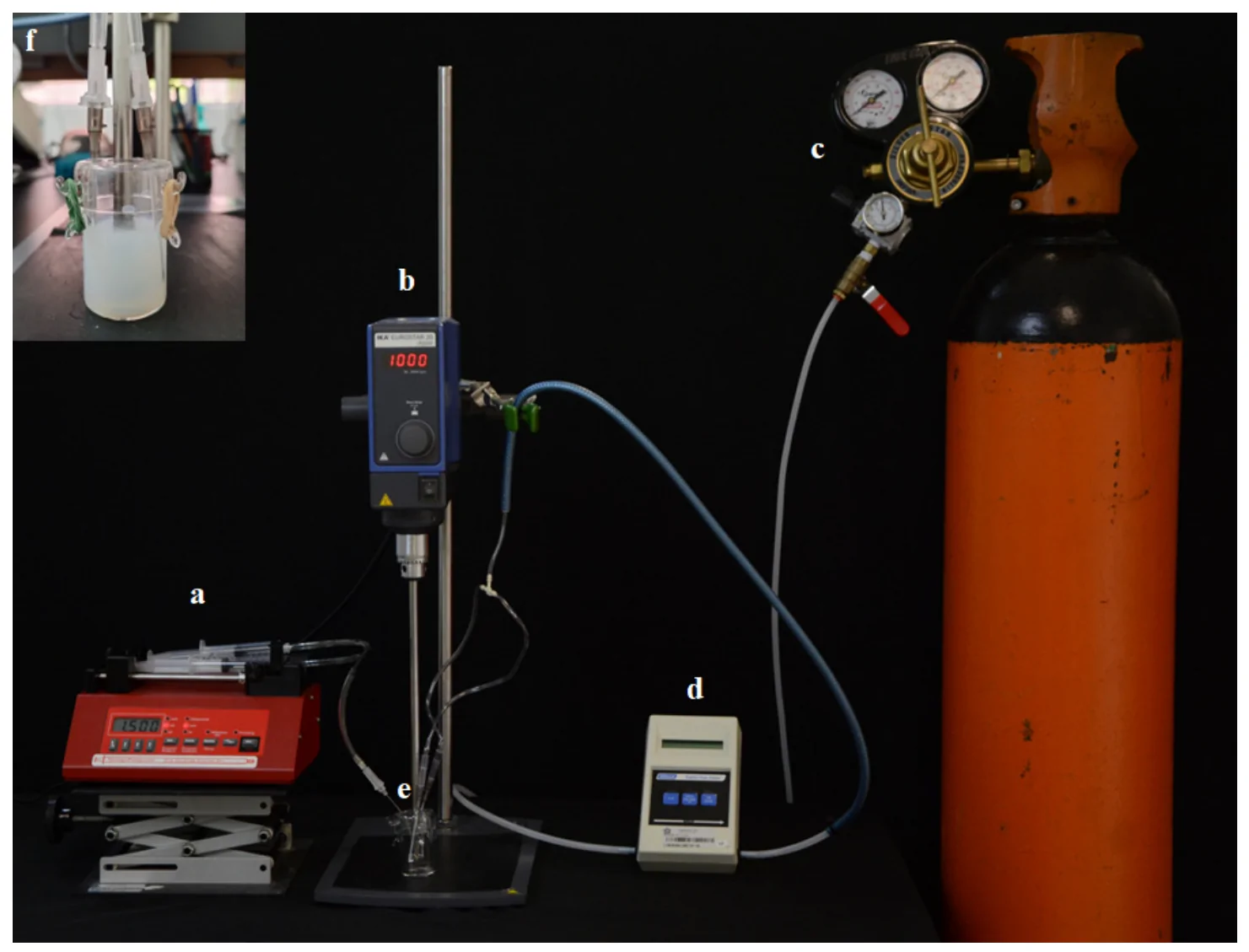
Reactor and drag system for BSA–SGI-1027 NP synthesis. (a) Ethanol administration by a double syringe array. (b) Agitation by internal propeller driven by external motor. (c) N_2_ injection from a pressurized tank. The caudal was reached by a double-pressure regulator. (d) Digital flow check. (e) Borosilicate glass reactor with a 50 mL capacity. (f) Detail of the injection of N_2_ during the crosslinking process of NPs.

In order to synthesize NPs, a reactor was manufactured to achieve a reproducible process and low-cost operation. We designed a 50 mL-capacity reactor with a removable cap featuring holes for a propeller, solution administration, and N_2_ injection (Figure 1). The ethanol addition (desolvation agent) was performed by an array of double syringes controlled by a syringe pump (a). The agitation was maintained at 1000 rpm throughout the process, avoiding stopping the agitator to add the desolvating agent or crosslinker (b). The N_2_ injection was regulated by a double regulator, first from 3000 to 50 psi and second from 50 to 5 psi (c), and was monitored by a digital flow check (d). The controlled flux of N_2_ allowed the ethanol to vaporize and spread around the propeller, creating a barrier against environmental impurities (e). The validation of NP synthesis is essential for the pharmaceutical industry; hence, three batches of each treatment were synthesized. The performance and recovery volume (7.5 mL) of the NPs were similar, provided that the N_2_ flux was maintained at 250 SCCM. The inset of Figure 1f shows a close-up view of the reactor during the NP synthesis; the appearance of the NP synthesis with glucose assisted with N_2_ is similar to the glutaraldehyde crosslinking process. In this case, there is no toxic gas formation from the glutaraldehyde, and the resulting suspension of NPs was completely dispersed in water. This glass reactor was manufactured in the glass-blowing workshop of the Chemistry Faculty of UNAM.

### 3.2. NP Morphology

**Figure 2:**
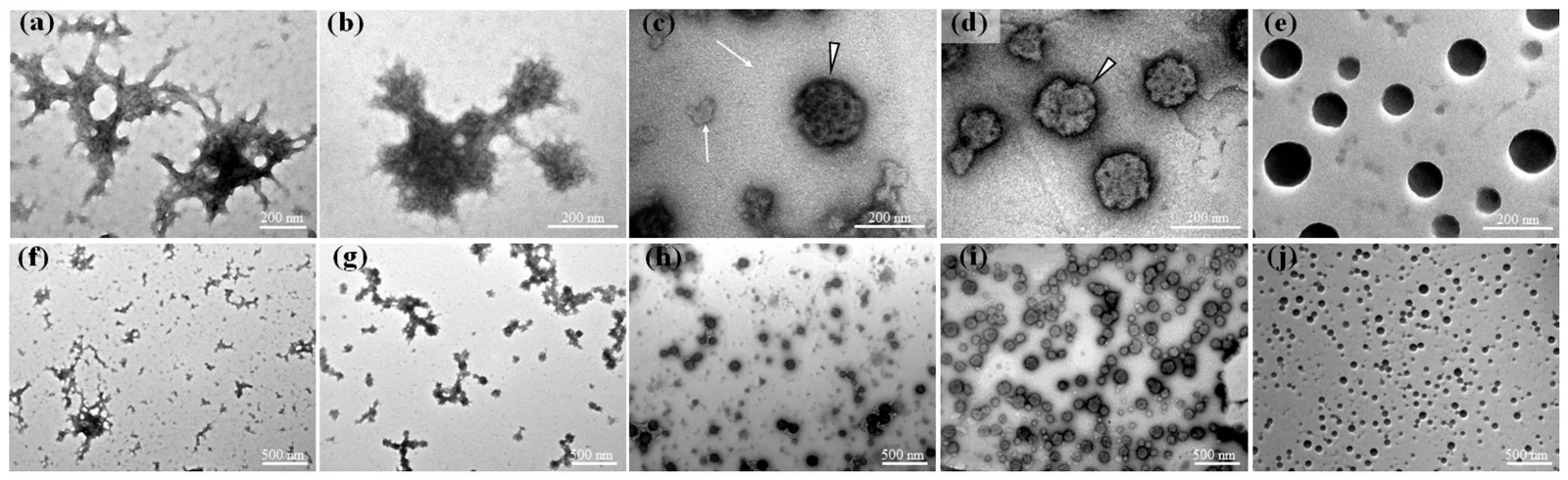
Morphology of the NPs visualized via TEM. High-magnification TEM micrographs revealed the morphology and nanostructuring of isolated NPs. (**a**) Control treatments without SGI-1027 with glucose but N_2_-lacking. (**b**) Control treatment without glucose but with N_2_. (**c**) Empty NP reveals irregular surface and formation of a crown around the NP (white arrow). (**d**) NPs with 0.05 mg of SGI-1027 show smoother surfaces and a decreased crown (white arrow). (**e**) NPs with 0.1 mg of SGI-1027 show a completely smooth and circular shape and no crown. (**f**–**j**) TEM micrographs at low magnification showing the distribution of NPs for different SGI-1027 concentrations. All TEM experiments were conducted at an 80 kV high-voltage (EHT), 140,000× (high magnification), 22,000× (low magnification), and 5 × 10^−3^ Pa (5 × 10^−5^ Torr) working pressure.

A robust method to evaluate the nanostructuration and morphology of NPs is TEM visualization, as described in previous reports [30,31,32]. In TEM images, intense contrast is associated with higher electron interaction, usually indicating a strong affinity for uranyl acetate, with free amino groups (–NH) and carboxyl groups (–COOH) of the BSA molecules [33]. For this study, NP morphology is an important parameter to evaluate, given the unreported crosslinking process, which occurred through the simultaneous action of glucose and assisted by the drag-solvent. The control treatments without SGI-1027 with glucose but N_2_-lacking showed precipitation of the BSA (Figure 2a). In this treatment, NP formation probably occurred after desolvation; however, crosslinking was not carried out, the NPs were disassembled during the washing process, probably due to instability of the condensed protein. A similar effect was observed in treatment without glucose but with N_2_, which showed coalesced reticulated structures formed by isolated NPs that lost shape during the washing process (Figure 2b). In both cases, the panoramic images showed similar structures to those observed at high magnification (Figure 2f,g). In fact, there is a synergistic effect between glucose and N_2_ that allowed the formation and stabilization of NPs. Therefore, both treatments were discarded for subsequent characterization. The NPs without SGI-1027 exhibited a different morphology compared with previously reported empty NPs [7,14,20,28,33]. The surface exhibited the formation of cavities (Figure 2c), which is probably due to faster ethanol evaporation induced by dragging with nitrogen gas. The morphology of the self-assembly of BSA molecules has not been reported or described before. Our NPs without SGI-1027 showed a “crown” structure around the NPs (Figure 2c, white triangle) and small agglomerations of BSA, which probably do not allow condensation on the NPs (Figure 2c, white arrows). In a panoramic image, the NPs showed similar characteristics in the surface and heterogeneity of size; moreover, they showed the presence of a “crown” around the nanostructures and small agglomerates of BSA (Figure 2h). Subsequently, at a concentration of 0.05 mg of SGI-1027, the NPs exhibited reduced surface texture and increased size. In addition, the “crown” decreased and the small agglomerates of BSA disappeared (Figure 2d, white triangle). In a panoramic image, the heterogeneous size of NPs was observed, along with an irregular morphology (Figure 2i). Finally, the treatment at 0.1 mg of SGI-1027 showed NPs with a circular shape and a slight decrease in size. The “crown” structure disappears, and the NPs showed intense staining, probably indicating changes in the polycondensation of the albumin molecules and increased exposition of functional groups (–NH) and (–COOH) of the BSA on the surface, indicating an increment of the drug load under the NPs (Figure 2e). Similar morphology was observed in BSA NPs stabilized with glutaraldehyde and heat [34,35]. The panoramic image showed a homogeneous morphology and a range of NP sizes (Figure 2j). These morphologies and size distributions are similar to those observed in previous studies in which NPs were loaded with high concentrations of hydrophobic drugs such as capsaicin [14].

### 3.3. NP Yield and SGI-1027 Encapsulation Efficiency

**Figure 3:**
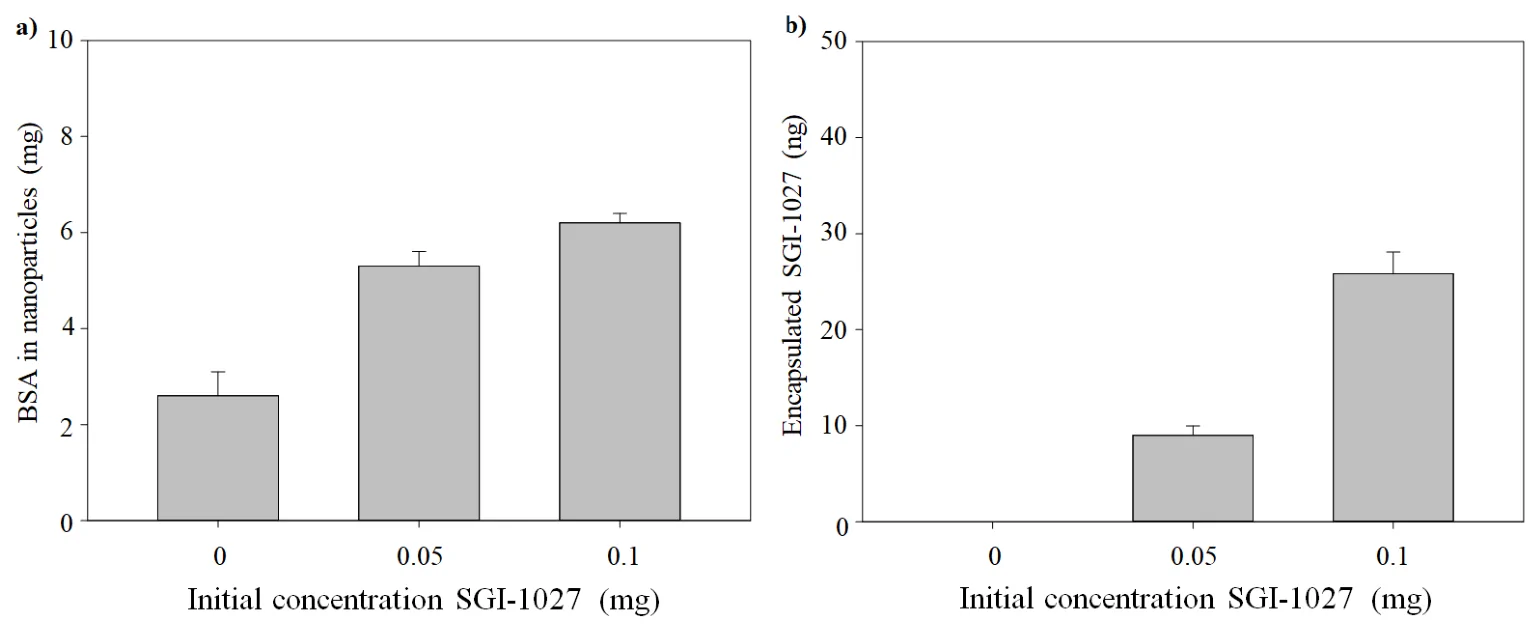
BSA and SGI-1027 content in NPs for different SGI-1027 doses. (**a**) Recovery of NPs after desolvation-assisted nitrogen and glucose crosslinking shows an increment as the SGI-1027 content in the formulation increased. (**b**) SGI-1027 encapsulated in NPs. Values are means of three experimental replicates ± standard deviations.

The yield of native BSA nanostructured into NPs increased when the SGI-1027 drug was included. In the NPs at 0 mg of SGI-1027, 2.6 mg (±0.5) of dried NP per milliliter was recovered. The yield of the NP without SGI-1027 was 20.19% ± 3.9 (Figure 3a). In contrast, a similar yield (22.6%) was reported by Sanchez-Marquez et al. [7] for BSA NPs loaded with a higher concentration of SGI-1027 and crosslinked with glutaraldehyde. For treatment of 0.05 mg of SGI-1027, an increase in BSA transformed into NPs was obtained; of every milliliter of NPs, 5.3 mg (±0.3) was recovered, representing a yield of 41.6% ± 2.6. For a dose of 0.1 mg of SGI-1027, the yield was 49.3% ± 1.71, while the recovery mass of NPs was 6.2 mg (±0.2) per milliliter of sample (Figure 3a). The one-way ANOVA revealed that differences in NP yields between treatments are statistically significant (α = 0.050, *p* ≤ 0.001), and a post hoc test, Student–Newman–Keuls, indicated that all the treatments are different (*p* < 0.05). A similar trend was reported by Sánchez-Segura et al. [14] at low doses of encapsulated capsaicin (0.26–10 mg), and at medium-to-high concentrations of capsaicin (8.45–25.35 mg), the yield decreased [20]. On the other hand, as expected, drug encapsulation efficiency (EE) increased with an increasing SGI-1027 concentration (Figure 3b). Namely, for treatment with an initial dose of 0.05 mg of SGI-1027, recovery was 0.010 (±0.001) mg of SGI-1027 of the total NP suspension, representing 21.5% for the encapsulated efficiency, while for 0.1 mg of initial SGI-1027, we recovered 0.023 (±0.004) mg of the total NP suspension, an increase of around 2.3-fold, and this reached a 23.2% encapsulated efficiency. The Kruskal–Wallis analysis revealed a difference between treatments, and a post hoc Student–Newman–Keuls test found a significant difference between empty NPs and NPs loaded with SGI-1027 (*p* < 0.05). However, no significant difference was detected (*p* > 0.05) between treatments at 0.05 and 0.1 mg of SGI-1027. The low efficiency of the encapsulated drug could be due to the process of desolvation agent addition (ethanol). In this study, we used dropwise addition at 1.5 mL/min, while Bartlett et al. [21] used a method of infusion addition of ethanol at 0.625 mL/min with a similar syringe pump. Bartlett et al. [21] found that ethanol infusion maximized reproducibility of the synthesis, and efficiency increased compared with dropwise addition. However, adapting this method to our reactor requires redesigning the double-array syringe administration (Figure 1a).

### 3.4. Morphometric Parameters

**Figure 4:**
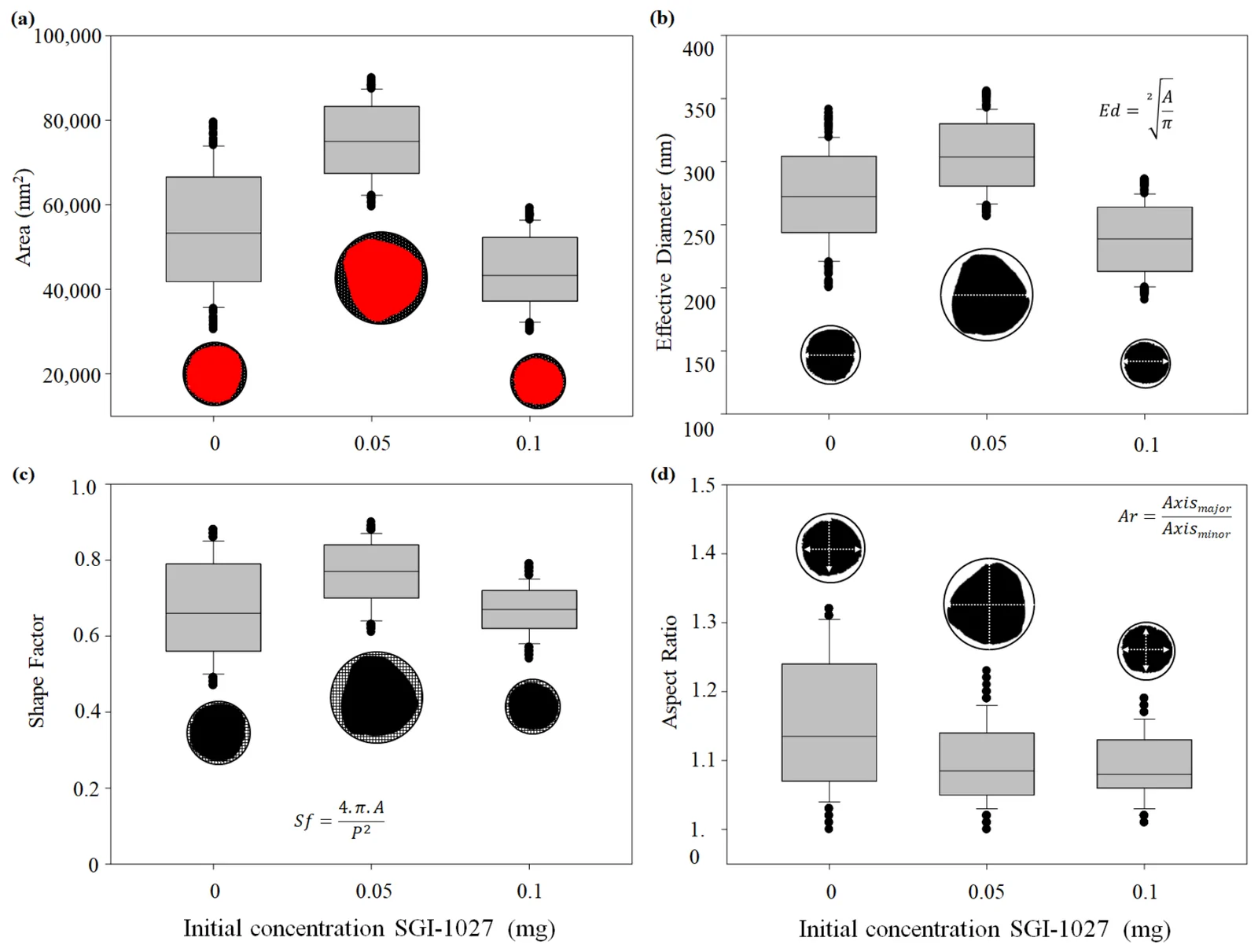
Morphometric parameters of SGI-1027 NPs. (**a**) The area of the NPs increased for 0.05 mg of SGI-1027 and decreased for 0.1 mg, relative to the control (NP at 0 mg). (**b**) The effective diameter of the isolated NPs increased at low concentrations of SGI-1027 and decreased at high concentrations. (**c**) The morphometric parameter of shape demonstrated the best circular shape of isolated NPs. The circular shape was lost in treatment at low concentrations (0.05 mg). However, the shape was recovered during treatment at high concentrations (0.1 mg). (**d**) The aspect ratio of NPs shifted from irregular dimensions (NP at 0 mg) to a more circular shape, with an equilibrium between the X-Y vectors as the concentration of SGI-1027 increased. Conditions were as follows: digital measurement of *n* = 180 NP images at 1376 × 1032 pixels and 8-bit compression; resolution set at 2.1 pixels/nm. Values are the means of three experimental replicates ± standard deviations.

Physicochemical properties of NPs are influenced by the morphological nanostructuration of some nanomaterials. However, a qualitative description is limited to describing the complexity of some dimensional characteristics. To establish statistical categories, TEM images can be analyzed using image analysis [36,37]. The area is a parameter that describes the dimensionality of a closed nanostructure, such as an NP, and its increase is not related to drug loading (Figure 4a). The estimated areas were 54,321.1 ± 14,300.3 nm^2^, 75,133.8 ± 8794.6 nm^2^, and 44,204.1 ± 8699.2 nm^2^ for 0, 0.05, and 0.1 mg of SGI-1027, respectively. The treatment with 0.05 mg of SGI-1027 showed a large area compared to other treatments (Figure 4a). However, statistical analysis showed a significant difference (*p* < 0.05) among all treatments. Regarding the *Ed*, it showed a similar trend as *A*; for 0 mg of SGI-1027 NP, *Ed* was 272.1 ± 36.6 nm; for 0.05 mg, it was 305.0 ± 28.6 nm, and for 0.1 mg, it was 239.1 ± 27.8 nm (Figure 4b). The *Ed* is the line of intersection between two points of the superimposed circumference in the image; this parameter was calculated from *A*; therefore, their behaviors were similar. However, the Student–Newman–Keuls test showed a significant difference (*p* < 0.05) between all treatments. On the other hand, *Sf* is a dimensionless parameter that describes the similarity to a circumference. The average *Sf* values were 0.66 ± 0.1, 0.76 ± 0.08, and 0.67 ± 0.06 for 0, 0.05, and 0.1 mg of SGI-1027, respectively (Figure 4c). This indicates that the NPs that showed higher bidimensional similarity to a circumference were from the treatment at 0.05 mg, followed by the treatment at 0.1 mg. The Student–Newman–Keul statistical analysis did not show a significant difference (*p* > 0.05) between empty NPs and 0.1 mg of SGI-1027, while the NPs at 0 mg exhibited a high degree of shape dispersion (Figure 4c). Finally, the *Ar* parameter describes the proportionality of object sizes in different dimensions; the calculated values were as follows: 1.15 ± 0.1, 1.10 ± 0.06, and 1.09 ± 0.05 for 0, 0.05, and 0.1 mg of SGI-1027, respectively. This indicates that the deviation from 1.0 (perfect circumference) is higher for the 0 mg of SGI-1027 NPs and tends to a quasi-circumference for treatment at 0.05 and 0.1 mg of SGI-1027 (Figure 4d). The Student–Newman–Keuls analysis shows a significant difference (*p* < 0.05) between empty NPs and NPs loaded with SGI-1027.

### 3.5. ζ-Potential, Hydrodynamic Diameter, and PDI

The ζ-potential is an important parameter for measuring the electrical state of a charged interface and affects the stability and dispersion of NPs in a liquid [38]. On the other hand, the hydrodynamic diameter is an estimate of the size of a particle in solution and differs from TEM image analysis. The hydrodynamic diameter is a correlation of the scattered intensity of a laser beam projected onto the NPs and the exposition time on a microsecond timescale due to the Brownian movement of the NPs. On this timescale, intensity fluctuations indicate the average of particle diffusion [39]. Both variables provide a panorama about transport, internalization, and retention in the target tissues and, thus, affect NP toxicity [38,40].

**Figure 5:**
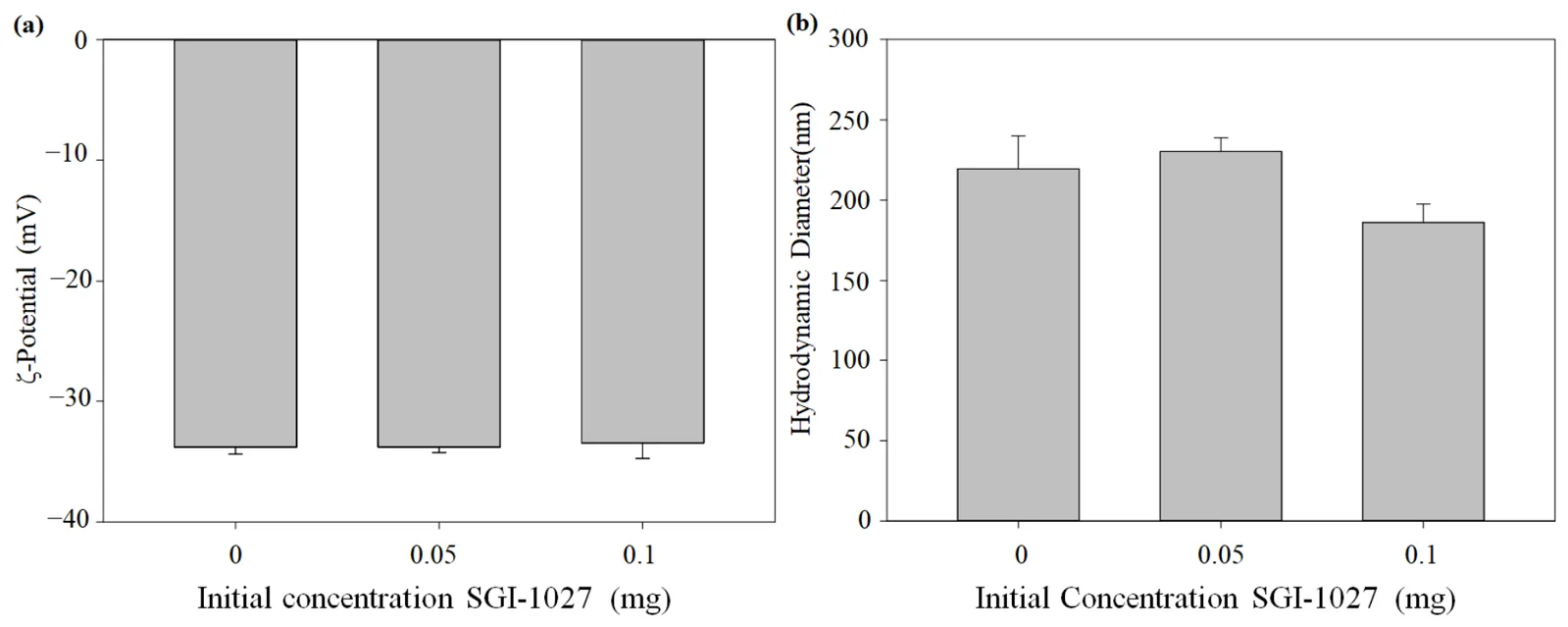
Effect of ζ-potential and hydrodynamic diameter in NPs of SGI-1027. (**a**) The electronegative surface of SGI-1027 NPs showed no significant changes. (**b**) A low concentration of SGI-1027 leads to increased particle size and aggregate formation, while a high concentration of SGI-1027 decreased the NP size. Values are means of three experimental replicates ± standard deviations.

A slight change in the ζ-potential, −33.8 ± 0.57, −33.47 ± 1.28, and −33.80 ± 0.46 mV, was observed in treatments of 0, 0.05, and 0.1 mg of SGI-1027, respectively (Figure 5a). The one-way ANOVA revealed that the differences in ζ-potential between treatments are not statistically significant (α = 0.050, *p* = 0.909); however, the ζ-potential values indicate that the NPs exhibited moderate electrostatic stability, ranging from ±30 to ±40 mV [38]. This parameter contrasts the ζ-potential values reported for HAS NP loaded with SGI-1027 and glutaraldehyde crosslinking [7]. However, it showed similar values to empty and BSA-capsaicin NPs formulated at a low concentration, according to a previous report, in which the surface electric charge was modified by the drying procedure and the use of post-crosslinking agents, such as sucrose [28]. The ζ-potential values reported here evidence the crosslinking activity of glucose on the surface of NPs.

On the other hand, the hydrodynamic diameter was estimated as follows: 219.5 ± 20.5, 230.3 ± 8.5, and 186.2 ± 11.4 nm at 0, 0.05, and 0.1 mg of SGI-1027, respectively (Figure 5b). There are significant differences (α = 0.050, *p* = 0.034) between all treatments of NPs in hydrodynamic diameter, which was revealed in the one-way ANOVA. In fact, TEM and DLS measurements yielded slightly different results, reflecting the dry condition and the internal core of the NPs [20]. However, there could be other factors influencing the differences between techniques, such as the shape, angle dependence, concentration, charge (polyelectrolytes and a double layer), contrast, particle aggregation, size-weighting (number vs. intensity weighted), small peak suppression, and radiation damage in TEM experiments [41]. The hydrodynamic diameter allows us to deduce the relation between particle size and physiological processes [42]. Finally, the polydispersity of the samples is an important parameter describing the NP size distribution. Several reports indicate that a PDI below 0.3 indicates low NP polydispersity. Therefore, values trending to 1.0 should be regarded as a greater distribution. For this study, the mean PDI values were 0.19 ± 0.09, 0.16 ± 0.15, and 0.21 ± 0.18 for 0, 0.05 and 0.1 mg of SGI-1027, respectively. The one-way ANOVA showed that differences in PDI between treatments were only marginally significant (α = 0.050, *p* = 0.049). These values indicate that the sample showed low polydispersity; however, in low-magnification TEM images, NPs of several sizes were distributed across short regions of interest (ROIs). As aforementioned, the PDI range was reported as an absolute limit. However, Pochapski et al. [38] propound that absolute PDI values below 0.1 can be considered as a low polydispersity for NPs. Therefore, our values showed low polydispersity, but it is possible to find NPs with several sizes.

### 3.6. Characterization of Molecular Interactions via FTIR

**Figure 6:**
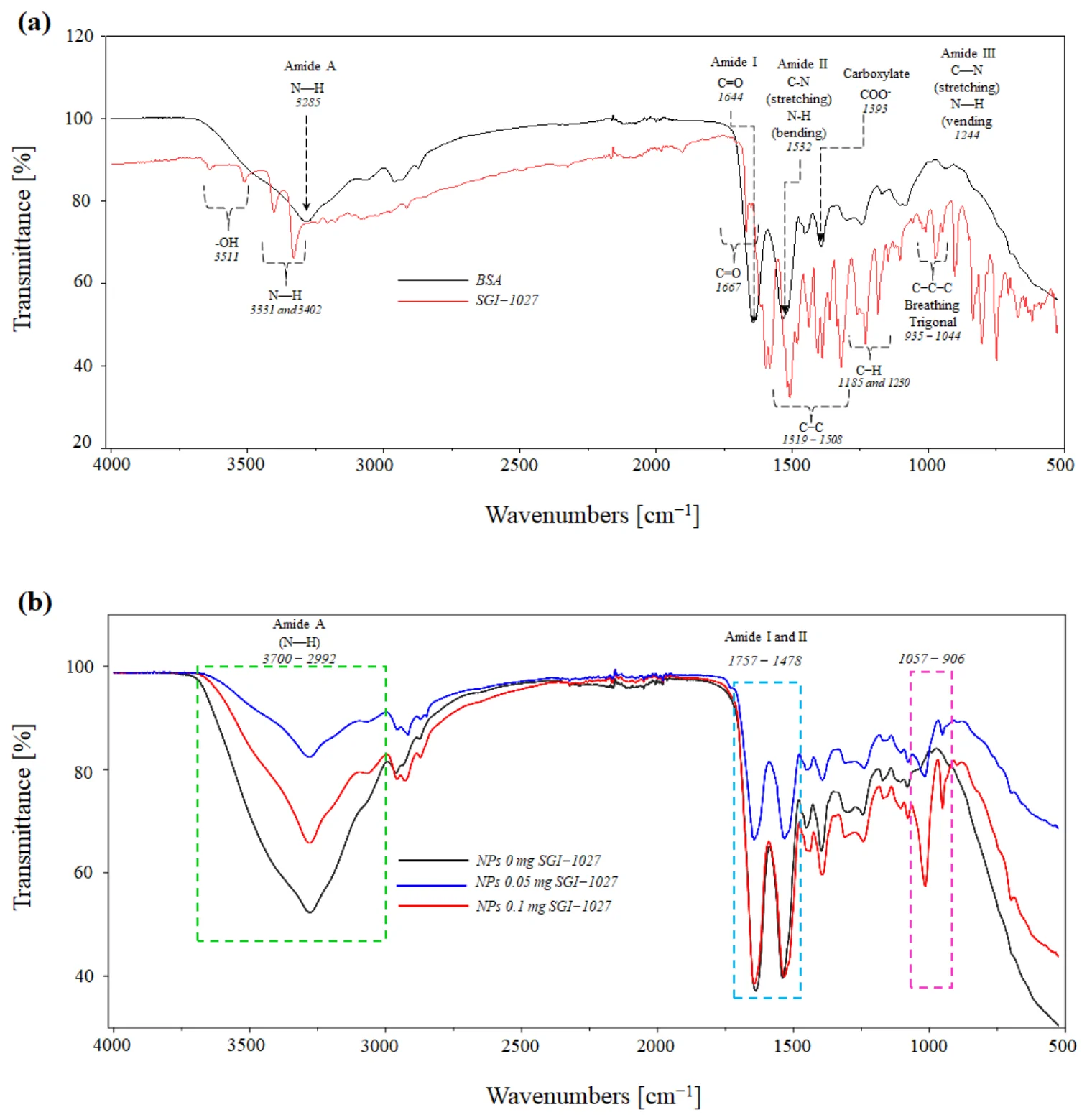
FTIR spectroscopy of SGI-1027 and BSA molecules and the effect of crosslinking on NPs. (**a**) FTIR spectra of SGI-1027 (red line) and BSA before desolvation-assisted nitrogen and glucose crosslinking (black line). (**b**) FTIR spectra of BSA loaded with SGI-1027 at 0, 0.05, and 0.1 mg of SGI-1027 (black line, blue line and red line, respectively) after desolvation-assisted nitrogen and glucose crosslinking. The fingerprint of the interaction between BSA and SGI-1027 was observed in the region from 1057 to 906 cm^−1^ that correspond to the C—C—C ring breathing and C—C—C trigonal bending peaks of SGI-1027 (dotted magenta rectangle). The spectral range for scanning was 4000-500 cm^−1^ in transmittance mode.

The native BSA and solid powder of SGI-1027 were analyzed to characterize the functional groups prior to supramolecular assembly and crosslinking. To elucidate the FTIR, the chemical structure of SGI-1027 was analyzed according to the chemical description previously mentioned. SGI-1027 is composed of 4-aminoquinoline, 4-aminobenzoic acid, 1,4-phenylenediamine, and 2,4-diamino-6-methylpyrimidine [10]. The chemical structure of SGI-1027 exhibited a 5-benzene-ring pattern, along with several functional groups (Figure 6a, red line). The benzene ring is commonly described as a broad band with five peaks from 1400 to 1650 cm^−1^. Although the benzene ring breathing and trigonal bending (C—C—C) exhibit characteristic vibrational activity at 995 and 1010 cm^−1^, respectively [43], for this study, the frequencies were found from 935 to 1044 cm^−1^. The 4-aminoquinoline ring has been associated with three carbon–carbon stretching bands near 1600 cm^−1^ (C—C) and five bands in the frequency of 1300 to1500 cm^−1^ [44]. In SGI-1027, the C—C stretching vibration of the benzene ring corresponds to the 4-aminoquinoline ring, which showed a series of peaks at 1319, 1362, 1388, 1405, 1439, 1481, and 1508 cm^−1^. However, the C=C stretching vibration cannot be associated with the peaks observed at 1500 cm^−1^, as suggested by Puviarasan et al. [43]. Previously, the weak frequencies at 1185 and 1230 cm^−1^ were matched with bending vibrations in plane C—H. The peaks observed at 3331 and 3402 cm^−1^ are attributed to N—H and NH_2_ symmetric (sym) and asymmetric (asym) stretching, characteristic of free 2-amino-5-methylpyridine [45]. With respect to the 1,4-phenylenediamine, the spectral fingerprint was found between 1380 cm^−1^ and 1600 cm^−1^ [46] under the SGI-1027 molecule; this group overlapped with the benzene rings of 4-aminoquinoline. The spectral fingerprint of the 4-aminobenzoic acid is attributed to aromatic compounds that are structurally similar to benzene. The C=O stretching vibration that was reported from 1600 to 1715 cm^−1^ [47] for this study was found at 1667 cm^−1^. Hence, CH-in plane bending and C—H-out of plane bending vibrations were reported from 1000–1400 cm^−1^. For this study, this group overlapped with the C—H functional groups of the 4-aminoquinoline ring. However, the carbonyl group and hydroxyl group vibrations are usually found between 3200 and 550 cm^−1^ [47]. For this study, a frequency of 3511 cm^−1^ was found (Figure 6a, red line).

The raw BSA was reported in previous reports [20,28]. The BSA showed a spectral fingerprint composed of several amide functional groups (Figure 6a, black line). The huge peak at 3285 cm^−1^ matched to amide A and corresponds to N–H stretching vibration [48], whilst the C=O stretching coincides with the amide I frequency at 1644 cm^−1^ [49]. An analogous peak at 1532 cm^−1^ was associated with amide II, and the frequency corresponds to C–N stretching and N–H bending resonance. The carboxylate functional group (COO-) was associated with the frequency at 1393 cm^−1^, although some authors have attributed it to CH_2_ groups [48,50]. Amide III matched the frequency observed at 1244 cm^−1^, corresponding to C–N stretching and N–H bending [28] (Figure 6a, black line).

BSA NPs loaded with SGI-1027 at 0, 0.05, and 0.1 mg showed some changes with respect to the spectrum of the original molecules. The frequencies between 3700 and 2992 cm^−1^ showed a modified peak (broad peak), with a high intensity at 3281 cm^−1^, matched with the N–H stretching vibration of amide A in BSA (Figure 6b, green rectangle), and overlaps with the N–H of 2-amino-5-methylpyridine and −OH group of SGI-1027 (Figure 6a). However, compared with previously reported results using albumin as the wall material and hydrophobic drug, the NPs with SGI-1027 did not show a progressive decrease in the magnitude of the NH amide A frequency (of BSA) with an increasing drug load (Figure 6b). The range between 1757 and 1478 cm^−1^ showed an analogous effect and was matched to an overlap effect between amide I and II bands of BSA and carbonyl linkage (C=O) and the alkane linkage (C–C) of the SGI-1027 (1667–1481 cm^−1^) (Figure 6b, cyan rectangle). This effect was observed in pazopanib-enzalutamide anticancer drugs encapsulated with BSA; the peaks of the free drugs were overlapped due to the no-covalent interaction with albumin [51]. The fingerprint of the interaction between BSA and the SGI-1027 was found in the region from 1057 to 906 cm^−1^, there are two peaks in this region corresponding to the breathing ring and trigonal bending (C—C—C) frequency of SGI-1027 (Figure 6b, magenta rectangle). These peaks showed a progressive increment in intensity relative to the initial SGI-1027 concentration added to the NP formulation.

### 3.7. Raman Spectral Analysis

**Figure 7:**
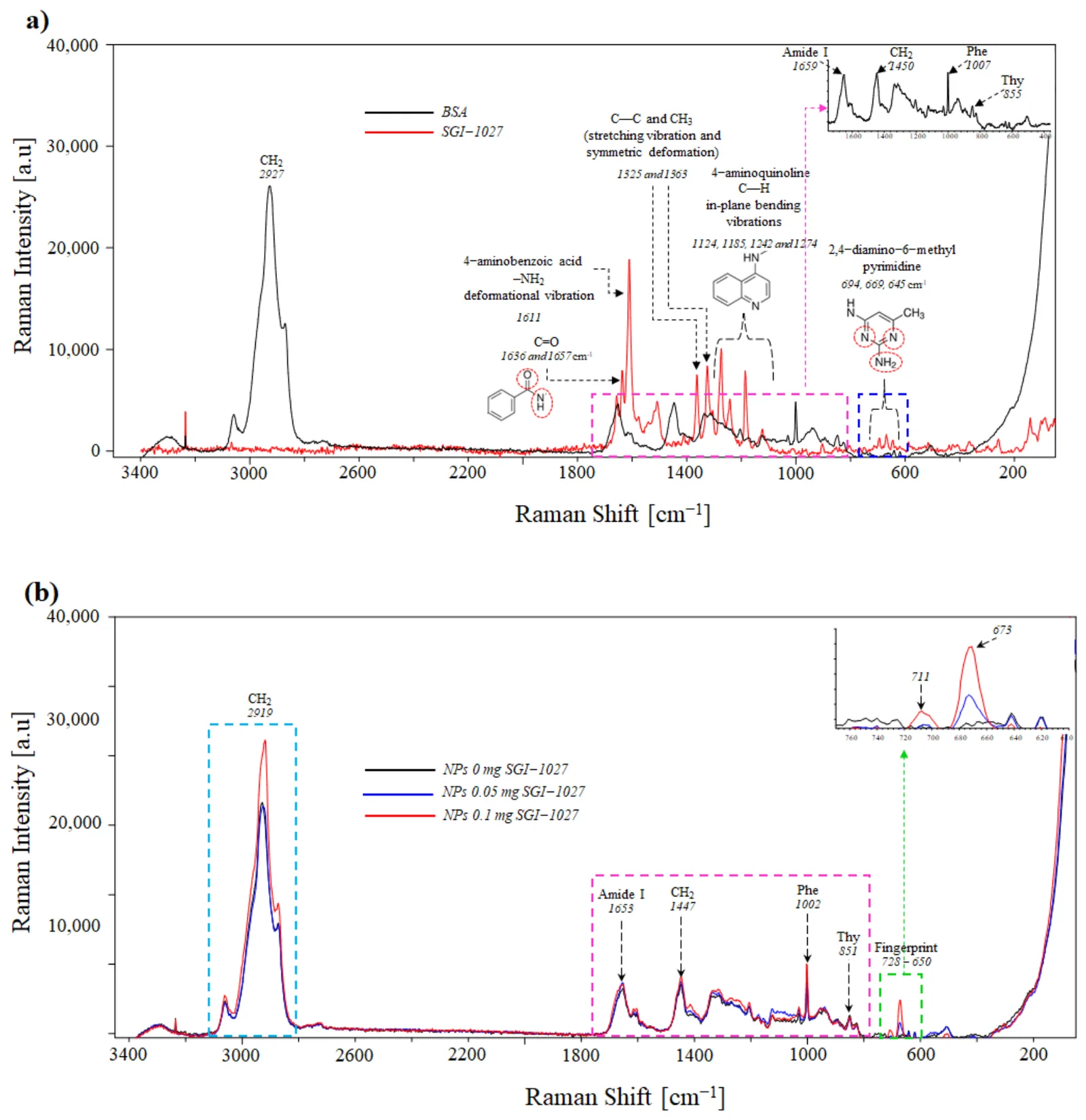
Raman spectroscopy of SGI-1027 and BSA molecules, and the effect of crosslinking on NPs. (**a**) Raman spectrum of SGI-1027 (red line) shows a fingerprint region comprising three peaks at 645, 669, and 694 cm^−1^ (dotted blue rectangle). The BSA fingerprint region shows the amide I band, the –CH_2_, and the hydrophobic region of phenylalanine (Phe) and tyrosine (Tyr) (dotted magenta rectangle and inset). (**b**) Raman spectra of BSA–SGI-1027 NP at 0, 0.05, and 0.1 mg of SGI-1027 (black, blue and red lines, respectively) after crosslinking. In the BSA NPs loaded with SGI-1027, the fingerprint showed two peaks: a short peak at 711 cm^−1^ and larger peak at 673 cm^−1^, which matches the fingerprint of SGI-1027, assigned to 2,4-diamino-6-methylpyrimidine.

Raman analysis shows slight similarity to FTIR due to molecular vibrational frequencies and the wavelength scales. However, as several authors have noted, Raman analysis is different from FTIR and provides complementary information about molecular interactions. On the other hand, to our knowledge, the Raman spectrum of SGI-1027 has not been published or described in any previous report. Therefore, based on the SGI-1027 FTIR description, the Raman spectrum was elucidated from its components. The SGI-1027 spectrum showed several bands, of which the fingerprint was composed of three peaks at 645, 669, and 694 cm^−1^; these spectral bands were assigned to 4−monosubstituted pyridines [52], which match with 2,4−diamino−6−methylpyrimidine of the SGI-1027 (Figure 7a, dotted blue rectangle). The pyrimidine molecule is composed of six units of the 1,3−diazine heterocycle containing two C=N functional groups. The second broadband corresponds to 4−aminoquinoline and is composed of 1124, 1185, 1242, and 1274 cm^−1^, which are matched to the C—H in-plane bending vibrations [43], followed by C—C stretching vibration and CH_3_ symmetric deformation found at 1325 cm^−1^ and 1363 cm^−1^, respectively (Figure 7a, dotted arrows). Finally, the last section of the bands corresponds to 4-aminobenzoic acid. In some reports, it has been established that the region 3500–3200 cm^–1^ was associated with primary amines (N–H stretching vibrations), and the asymmetric –NH_2_ stretching vibration was assigned from 3500 to 3420 cm^–1^, and the symmetric –NH_2_ stretching corresponds to 3420 to 3340 cm^–1^. In the 4−aminobenzoic acid molecule, the amino group is not involved in hydrogen bonding formation. Hence, the primary amine of the 4−aminobenzoic acid apparently showed a linked function between 4−aminoquinoline and 2,4−diamino-6-methylpyrimidine in the core of the SGI-1027, causing moderately strong in-plane –NH_2_ deformational vibration in the short range 1678–1564 cm^–1^. It is probable that the strong band found at 1611 cm^–1^ was matched to the amino group deformation mode and is influenced by C=O stretching peaks at 1636 and 1657 cm^−1^, both functional groups of 4-aminobenzoic acid [53].

The BSA Raman spectral description was reported by Sánchez-Segura et al. [20]; namely, it exhibited a strong peak from 3022 to 2821 cm^−1^ and the fingerprint band from 2000 to 400 cm^−1^. Socrates [52], Chikashi and Kazufumi [54], and Sánchez-Segura et al. [20] attributed the strong peak at 2927 cm^−1^ to the C—H linkage vibration of the methyl and methylene terminals groups (Figure 7a). However, large bands of absorption frequencies were attributed to −CH_3_ or −CH_2_, due to the protons directly attached to C, and the effect of electronegative groups or atoms is to increase the C—H absorption frequency. Nevertheless, in the BSA fingerprint, the frequency at 1659 cm^−1^ matched the amide I, and the –CH_2_ stretching frequency was found at 1450 cm^−1^. The phenylalanine (Phe) hydrophobic region was found at 1007 cm^−1^ of frequency and was associated with angular phenyl ring bending vibrations, whilst the linked Y5–Y6 side-by-side frequency at 855 cm^−1^ was associated with the Cα–Cβ of the amino linked to the aromatic side chain orientation of the phenyl ring linked to the tyrosine (Tyr) main chain backbone (Figure 7a, magenta rectangle) [55,56,57]. It is important to emphasize that the molecular interactions of BSA are attributed to its secondary and tertiary folded structure and the microenvironment of each functional group [54]. The desolvation and crosslinking proposed in this work likely led to different nanostructuration through the reorganization of these structures.

The NPs at 0, 0.05, and 0.1 mg of SGI-1027 showed changes in the spectra of the original molecules (Figure 7b). The first band was found between 3030 and 2819 cm^−1^, with a maximal intensity at 2919 cm^−1^, and matched to the CH_2_ (Figure 7b, cyan rectangle); there is an increment of intensity for the NPs at 0.1 mg of SGI-1027, likely due to the accumulation of drug. The second band was found from 1712 to 796 cm^−1^; the peaks observed correspond to functional groups of BSA. The peaks at 1653, 1447, 1002, and 850 cm^−1^ were identified as amide I, CH_2_, phenylalanine, and tyrosine, respectively (Figure 7b, magenta rectangle). In this band, no changes were observed in the peaks of the NPs relative to native BSA. However, the fingerprint of the SGI-1027 encapsulated with BSA was found at 728–650 cm^−1^. This band showed two peaks: a low peak at 711 cm^−1^ and a larger peak at 673 cm^−1^. This band matches the fingerprint of SGI-1027, which was assigned to 2,4-diamino-6-methylpyrimidine with three peaks at 694, 669, and 645 cm^−1^ (Figure 7a, blue rectangle). This band was not observed in the treatment at 0 mg of SGI-1027 but appeared at 0.05 mg of SGI-1027 and increased in intensity at 0.1 mg of SGI-1027 (Figure 7b, green rectangle and inset).

### 3.8. SDS-PAGE of the NPs

**Figure 8:**
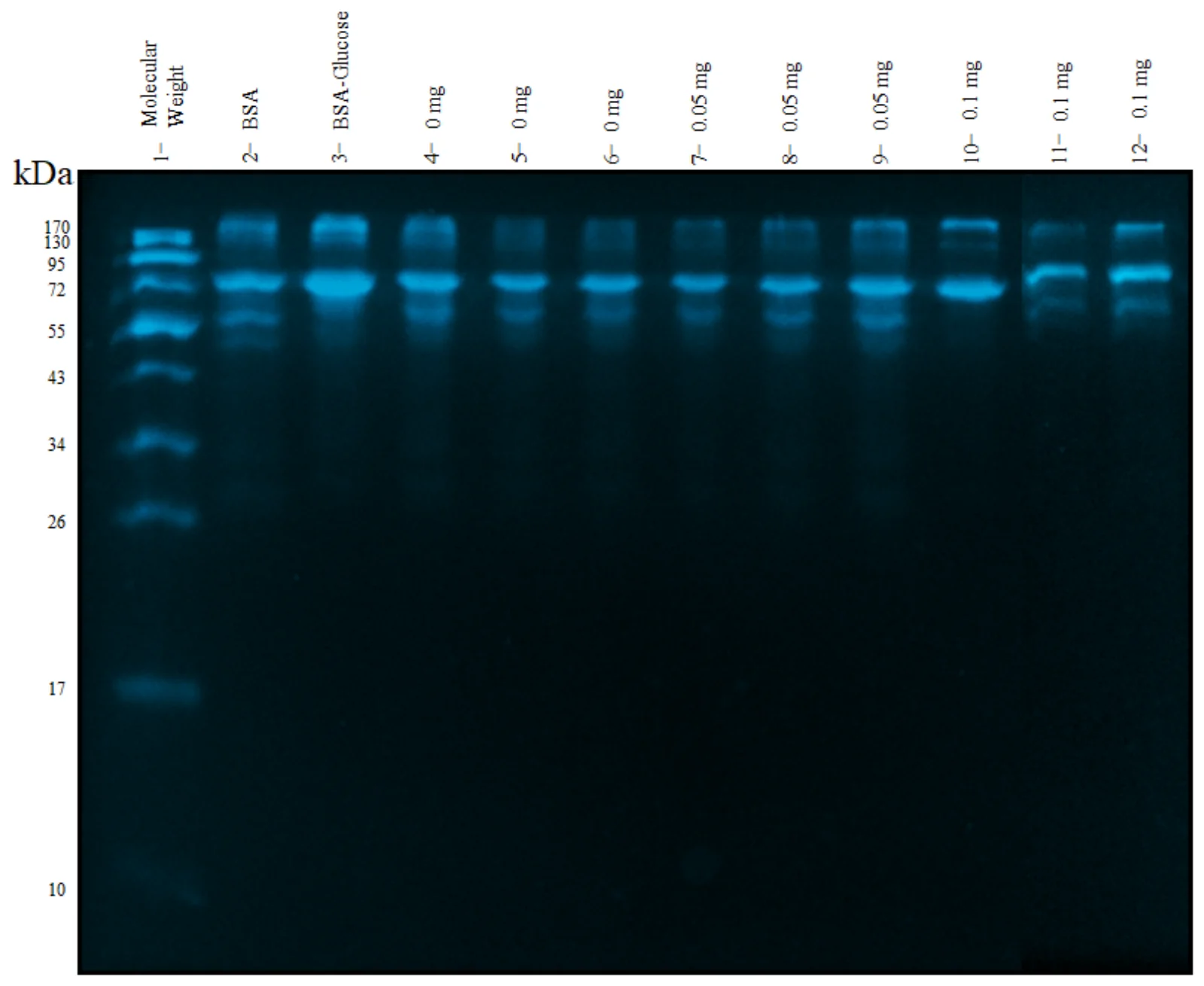
SDS-PAGE of native BSA and disassembled BSA NPs loaded with SGI-1027. Lane 1 shows the molecular weight (MW) ladder, from 10 to 170 kDa. Lane 2, native BSA corresponding to the electrophoretic pattern at 66 kDa. Lane 3, BSA and glucose without desolvation and crosslinking. Lanes 4–6 correspond to the BSA NPs at 0 mg. Lanes 7–9 correspond to NPs loaded with a low concentration of SGI-1027. Lanes 10–12 correspond to NPs loaded with SGI-1027 at high concentrations.

The molecular size and purity of the proteins have been characterized via SDS-PAGE [58]. In nanobiotechnology, the SDS-PAGE has been applied to characterize stability, post-synthesis nanostructuration, and contrast of the effect of several crosslinking compounds on BSA NPs [20,28,59]. To demonstrate the biosecurity of the residual BSA and the effect of crosslinking, SDS-PAGE was performed. We loaded the gel with native BSA, native BSA with glucose (without the solvent drag), and treatments of NPs at 0, 0.05, and 0.1 mg of SGI-1027 and then separated them via electrophoresis. The native BSA showed a defined band from 66 to a 72 kDa range (Figure 8, lane 2). The lane corresponding to native BSA with glucose (without the solvent drag) showed a change in the intensity pattern in the 55–90 kDa region (Figure 8, lane 3), probably due to an increase in the BSA molecular weight (MW) resulting from the partial glycation and formation of AGEs. A similar electrophoretic pattern was reported by Ledesma-Osuna [60]. The NPs at 0 mg of SGI-1027 showed similar electrophoretic patterns (Figure 8, lanes 4, 5, and 6) and a slightly intensified band in the region 66–72 kDa, probably due to the quantity of BSA recovered in the sample preparation. A similar effect was observed with treatment at 0.05 mg of SGI-1027; the albumin molecules disassembled from the NPs showed a similar mass with respect to native BSA. The treatment at 0.1 mg of SGI-1027 showed changes between the first sample (Figure 8, lanes 10) and repetitions (Figure 8, lanes 11 and 12). The first sample showed a first band between 170 and 180 kDa, with the second predominantly at 66–72 kDa, which corresponds to the native albumin. While experimental repetitions showed a thickness band at 90 kDa, this indicated that the albumin was modified. In addition, the third treatment repetition at 0.1 mg (Figure 8, lane 12) showed a band at 180 kDa. The formation of higher-MW peptides contrasts the results of different crosslinking methods used to stabilize the NPs. Carriles et al. [28] found that glutaraldehyde-crosslinked BSA–capsaicin NPs did not disassemble when exposed to SDS-PAGE denaturing agents such as SDS, heat, and β-mercaptoethanol. While Sánchez-Segura [20] used heat as a crosslinking agent, they did not report the formation of disassembled proteins of a higher molecular weight.

### 3.9. Cellular Uptake and Secreted Pro-Inflammatory Cytokine Profile

**Figure 9:**
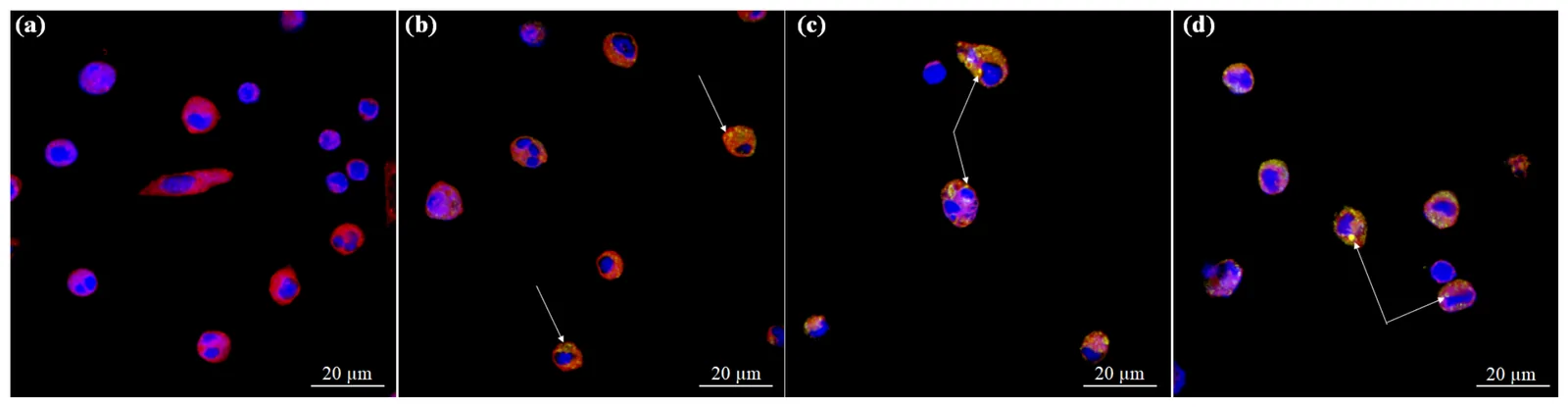
Cellular uptake of SGI-1027 NPs. (**a**) Control macrophage administered water; yellow fluorescence was not detected. (**b**) Macrophage cultures administered BSA NPs at 0 mg showed low internalization of the NP around the cell membrane and cytoplasm (with arrows). (**c**) At 0.05 mg of SGI-1027, the internalization of stained NPs increased and was located near the nuclear membrane (white arrows). (**d**) Treatment at 0.1 mg of SGI-1027 improved internalization and increased its presence around the nuclear membrane (white arrow).

**Figure 10:**
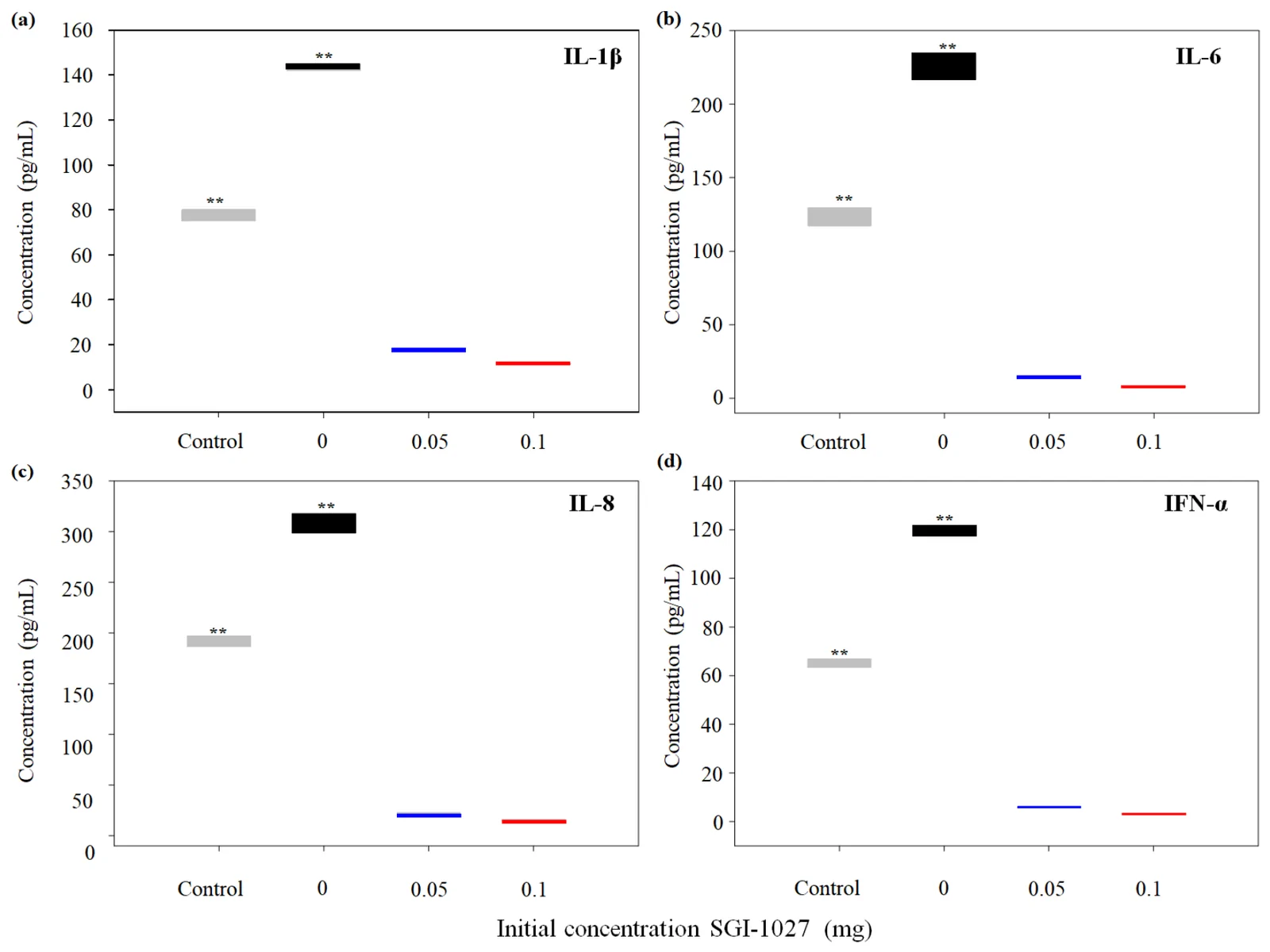
Profile of secreted cytokines in BSA NP-challenged mouse peritoneal macrophages. (**a**) Interleukin IL-1β. (**b**) Interleukin IL-6. (**c**) Interleukin IL-8. (**d**) IFN-α. In all graphs, the grey box represents the control, the black box represents water (negative control), the blue box represents 0.05 mg, and the red box represents 0.1 mg of SGI-1027. Asterisks mark the statistical significance of the difference with SGI-1027-loaded NPs (*p* < 0.01, ANOVA and Scheffe’s post hoc).

To confirm cellular uptake by macrophages, NPs stained with eosin Y were administered to MPMs. Water was used as a control. After the incubation time, the macrophages that were administered water showed two morphological forms: circular shape and elongated shape [61]. In both types of macrophages, a nucleus (in blue) and a cell membrane (in red) were identified (Figure 9a). Additionally, no yellow fluorescence was detected, demonstrating that MPMs do not exhibit autofluorescence. In MPMs challenged with NPs at 0 mg of SGI-1027 stained with eosin Y, internalized fluorescence was observed around the cell membrane and cytoplasm (Figure 9b, white arrow). MPMs showed changes, including a decreased size and nuclear fragmentation. We failed to observe cells with an elongated shape (Figure 9b). In MPMs challenged with NP loaded with 0.05 mg of SGI-1027, an increase in fluorescence internalization was observed. However, in this treatment, the intracellular distribution differed from the one of NPs at 0 mg, as fluorescence was additionally observed in proximity of the nucleus (Figure 9c, white arrow). Together with previous observations that BSA NPs localize near the nucleus, our data indicate that BSA NPs synthesized without glutaraldehyde as a crosslinker exhibit good capacity for internalization [62]. MPMs challenged with 0.1 mg of SGI-1027-loaded NPs showed a similar level of internalization to the aforementioned study. The internal distribution in this treatment encompassed all cells; however, the accumulation occurs at the nuclear border, probably attached to the nuclear membrane (Figure 9d, white arrow). The morphology of the macrophages was predominantly circular. Additionally, challenge with BSA NPs loaded with SGI-1027 resulted in dramatically low levels of all of the assessed pro-inflammatory cytokines (IL-1β, IL-6, IL-8, and IFNα) in the culture medium, compared to water (negative control) or void NPs (Figure 10). Notably, secreted cytokines were higher in the presence of void NPs compared to the control (*p* < 0.05 in all cases; ANOVA and Scheffé post hoc) (Figure 10).

## 4. Discussion

We aimed at better understanding the mechanism underlying the synergistic effect between glucose and N_2_ (solvent drag) during protein nanostructuration via self-assembly. Molecular self-assembly can be described as a spontaneous nanostructure-formation process driven by the synergistic effect of several forces, such as noncovalent linkages that include hydrogen bonding, π–π, electrostatic, hydrophobic, and van der Waals force interactions [63]. Nanostructuration via coacervation based on self-assembly is a process occurring based on the intrinsic interaction of macromolecules in solution. The result is differentiation into a coacervate phase and a dilute phase. A limited set of variables, such as the pH, ionic strength, or temperature, controls the process [17,64]. That differs from other encapsulation methods such as emulsification, where BSA is dissolved in the aqueous phase, and desolvation is carried out with ethanol (organic phase) under hydrostatic pressure via stirring; this process is known as the first emulsification step. Nanostructuration takes place in the second emulsification step, consisting of the extraction or evaporation of the organic solvent. This process was previously reported during enzyme immobilization, in which undesirable microparticle formation was observed following the second emulsification step [65]. In our study, we propose that the increased ethanol gradient creates an interface between the aqueous and organic phases, accompanied by a change in the native BSA protein structure, with the N isoform shifting from α-helix (0% to 50% ethanol) to the albumin F isoform with the predominance of β-sheets at 60% to 90% ethanol, and reconstituting it to a changed α-helix (albumin unknown isoform) at 90% and 99% ethanol [66]. The organic phase of ethanol allows exposure of the hydrophobic sites of α-helix side chains and weakens long-range interactions, causing destabilization among α-helices. Further, the translation from a highly polar solvent, such as water, to a nonpolar solvent environment, such as ethanol, causes a lower dielectric effect in the BSA via the large solvation of hydrophobic side chains [14]. During desolvation, the BSA becomes insoluble and condenses into self-assembled spherical aggregates; their shape is determined by the propeller geometry and the stirring velocity in the reactor; both parameters produce a nanostructured self-assembly. The stabilization of the NPs was achieved via evaporation of the solvent phase and a gradual, lower transition to the aqueous phase. This shift from a hydrophobic to a hydrophilic medium causes long-range interactions that stabilize peptide–peptide hydrogen linkages in BSA molecules during NP formation. As BSA nanostructuration was obtained, lysine–arginine crosslinking with glucose resulted in the formation of a glucosepane [21]. It is assumed that glucose-mediated crosslinking is initiated by condensation of its aldehyde group with lysine amino groups, producing early Schiff-base intermediates that can subsequently participate in glycation-derived crosslinks. Notably, glucose crosslinking of BSA creates undesirable products such as advanced glycation end products (AGEs), as confirmed via our SDS-PAGE data (Figure 8, lane 3). However, oxidation and AGE formation should be prevented by the immediate use of N_2_ after glucose addition. Sustained N_2_ injection during solvent evaporation causes soluble drugs (in water) and proteins to migrate to the aqueous phase, concentrating them at the microparticle surface and producing a ‘burst’ effect, which is often undesirable [67]. In our NPs, the process of solvent evaporation was carried out with a flux (250 CCMM) over a prolonged time (7 h), decreasing the possibility of adverse effects. The results show that nitrogen-drag-assisted crosslinking with glucose is reproducible across batches of BSA-loaded SGI-1027 NPs. At any rate, warranted future studies include the use of efficient nozzles for the dispersion of N_2_ into the reactor and by probing different rates of flux. With respect to cellular uptake, we found that the BSA-loaded SGI-1027 NPs showed a marked capacity to be internalized by mouse peritoneal macrophages and were localized around the cell membrane and cytoplasm and, in the case of 0.1 mg-BSA-loaded SGI-1027 NPs, around the nuclear membrane. This contrasts Márquez-Sánchez et al. [7], in which NP internalization improved through functionalization with a fusion peptide (FLAGPP1). S-HSANP-FLAGPP1 increased internalization, which was observed in cytoplasmic droplet-like structures and nuclear localization. Furthermore, SGI-1027-loaded NPs induced a clear decrease in secreted pro-inflammatory cytokines. Although encouraging for potential in vivo applications, our data are limited to macrophage internalization and inflammatory responses; therefore, we cannot conclude whether BSA NPs loaded with SGI-1027 NPs would affect other cell types relevant to atherosclerosis, such as endothelial or smooth muscle cells. Crucially, any effects on the DNA methylome will have to be assessed in the vascular wall and non-vascular tissues. Studies using relevant animal models and, if possible, preclinical human trials will be needed to test that hypothesis. In particular, further studies are warranted to define whether the apparent increase in cytokine secretion by void NPs in mouse peritoneal cells is related to the presence of advanced glycation end products resulting from glucose crosslinking or any alternative mechanism [68]. In summary, this hybrid method of coacervation–emulsification-assisted by drag solvent evaporation is an alternative to the encapsulation of thermolabile hydrophobic drugs without the use of chemical crosslinking agents.

## 5. Conclusions

We document a simple, glutaraldehyde-free and heat-free process for BSA crosslinking to obtain NPs loaded with the DNA methylation inhibitor drug SGI-1027. We found a synergistic effect between glucose and N_2_ (solvent drag) for protein self-assembly-nanostructuration under our conditions. NP morphological, morphometric, and physicochemical parameters were improved or comparable to counterparts obtained via crosslinking with glutaraldehyde or heat. However, we found that increasing the concentration of SGI-1027 in the formulation yielded results similar to those of glutaraldehyde-stabilized NPs. FTIR and Raman showed spectral fingerprint of SGI-1027 within NPs, thus assuring effective drug encapsulation. Regardless of the SGI-1027 concentration, the NPs preserve the capacity to disassemble to individual BSA molecules. Overall, our results showed that nitrogen-drag-assisted crosslinking with glucose offers advantages over conventional batch techniques for NP synthesis in reproducibility, property tunability, and scalability. Finally, SGI-1027-loaded NPs showed a marked capacity to be internalized by mouse peritoneal macrophages, where they localized around the cell membrane and cytoplasm and adhered to the nuclear membrane, and decreased the secretion of pro-inflammatory cytokines. This exploratory work supports the idea of green manufacturing, biosafety, and low-cost compared with aldehyde crosslinking.

## Data Availability

The original contributions presented in this study are included in the article. Further inquiries can be directed to the corresponding authors.

## Abbreviations

The following abbreviations are used in this manuscript:

A	Area
AGEs	Advanced glycation end products
Ar	Aspect ratio
ATR	Attenuated total reflectance
BSA	Bovine serum albumin
CANTOS	Canakinumab anti-inflammatory thrombosis outcome study
CBB	Coomassie brilliant blue
CICUAL	Institutional care and use of laboratory animals committee
COLCOT	Colchicine cardiovascular outcomes trial
CO_2_	Carbon dioxide
CVD	Cardiovascular disease
DLS	Dynamic light scattering
DMSO	Dimethyl sulfoxide
DNA	Deoxyribonucleic acid
dpi	Dots per inch
DW	Deionized water
Ed	Effective diameter
EHT	Extra high tension
EF	Encapsulation efficiency
FTIR	Fourier transform infrared spectroscopy
g	g force
HAS	Human albumin serum
HPLC-UFLC	High-performance liquid chromatography–ultra-fast liquid chromatography
IFNα	Interferon α
kDa	Kilodalton
LoDoCo2	Low-dose colchicine 2
min	Minutes
mL	Milliliter
MFI	Median fluorescence intensity
mg	Milligram
MPM	Mouse peritoneal macrophages
MW	Molecular weight
N_2_	Nitrogen gas
Ng	Nanogram
NP	NP
PB	Phosphate buffer
PDI	Polydispersity index
RGB	Red, green, blue
ROIs	Regions of interest
PP1	Protein phosphatase-1
rpm	Revolutions per minute
SA-PE	Streptavidin-phycoerythrin
SCCM	Standard cubic centimeters per minute
SDS-PAGE	Sodium dodecyl sulphate–polyacrylamide gel electrophoresis
Sf	Shape factor
TEM	Transmission electron microscopy
TIIF	Tagged image file format
UNAM	Universidad Nacional Autónoma de México
UV	Ultraviolet
